#  Association Study of Xenobiotic Detoxication and Repair Genes with Malignant Brain Tumors in Children 

**Published:** 2010

**Authors:** L.E. Salnikova, N.I. Zelinskaya, O.B. Belopolskaya, M.M. Aslanyan, A.V. Rubanovich

**Affiliations:** Vavilov Institute of General Genetics, Russian Academy of Sciences; Federal State Center “Russian Scientific Center of Roentgenoradiology”; Lomonosov Moscow State University

**Keywords:** gene polymorphism, malignant brain tumors in children, genes of xenobiotic detoxication, DNA repair genes

## Abstract

This study presents the results of research on DNA polymorphism in children with malignant brain tumors (172 patients, 183 in the control group). Genotyping was performed using an allele-specific tetraprimer reaction for the genes of the first (*CYP1A1 *(2 sites)) and second phases of xenobiotic detoxication (*GSTM1, GSTT1, GSTP1, GSTM3*), DNA repair genes*XRCC1, XPD*(2 sites),*OGG1*, as well as*NOS1*and*MTHFR.*The increased risk of disease is associated with a minor variant of*CYP1A1*(606G) (p = 0.009; OR = 1.50) and a deletion variant of*GSTT1*, (p = 0.013, OR = 1.96). Maximum disease risk was observed in carriers of double deletions in*GSTT1-GSTM1*(p = 0.017, OR = 2.42). The obtained results are discussed in reference to literary data on the risk of malignant brain tumor formation in children and adults.

##  Introduction 

The causes behind the formation of malignant tumors of the central nervous system (CNS) in children, of which 80% are cerebral tumors, are unknown. Risk factors for this type of pathology include inherited susceptibility and the effects of irradiation. Several genetic syndromes, such as the Li-Fraumeni syndrome, Turcot syndrome, neurofibromatosis, and tuberous sclerosis, are known to cause CNS tumors. Moreover, there are families with an increased risk of cerebral tumor formation. For instance, a population cohort from Utah (USA) and a tumor register, which was created based on data from this cohort, indicate the importance of the inheritance factor in most common malignant diseases of the brain in adults (astrocytomas and glioblastomas) [1]. Studies of the Swedish tumor register indicate that first-degree relatives are 2 to 3 times more likely to develop a brain tumor of the same histopathological type as their probands [2]. The offspring of people who had a brain tumor in their childhood are twice as likely to develop a similar tumor [3], the same being true for such a patient’s siblings, especially before the age of 5.

Relatives of patients with malignant diseases of the brain are also at risk of developing other oncological conditions. First-degree relatives of glioma patients have an increased standardized incidence ratio (SIR - ratio between the number of observed cases and the expected number) for developing any type of oncopathology (SIR = 1.21), especially before the age of 45 (SIR = 5.08). Relatives of glioma patients most often develop brain tumors (SIR = 2.14), melanomas (SIR = 2.02), and sarcomas (SIR = 3.83) [4].

Brain tumor incidence is now rising in the majority of highly developed countries, especially among children younger than 5 [5]. The role of environmental factors in childhood carcinogenesis, in general and in the CNS tumor development risk, is under investigation. An association has been established between *in utero* ionizing radiation and the risk of developing leucosis and other tumors in childhood [6]. Another such association has been observed for women using diethylstilboestrol during pregnancy and the risk of their daughters developing clear-cell vaginal adenocarcinoma [7]. It has also been shown that brain tumor development in offspring is often associated with parental occupational hazards, such as pesticides [8] or herbicides [9]. The association between maternal diets and the chance of their offspring developing brain tumors has also been researched. The most detrimental factors were found to be high amounts of nitrosamines, widely used for preserving meat and sausage products, as well as large amounts of fat [10, 11]. Transplacental carcinogens of alkyl-nitroseureas are highly carcinogenic in relation to rat brain tumors [12]. Children with excessive [14] or insufficient [13] birth weight, as well as children with an excessive head circumference (OR = 1.27 for every centimeter of excess after stratification of the cohort for sex, weight and height of the newborn) [15], and children whose mothers have had miscarriages in their anamneses are also at higher risk of developing brain tumors [16]. Intensive smoking (> 10 cigarettes a day) during pregnancy is also among the risk factors contributing to CNS tumors in the offspring [13].

If hereditary syndromes associated with the risk of malignant tumor formation in the nervous system are absent, then genes with low penetrance take on the role of genetic risk factors [17]. Even though the structure of neuro-oncological disease incidence in adults and children differs considerably [18, 19], it is the study of children with sporadic tumors that allows for the effective identification of genetic susceptibilities, as compared to studies of adults. The higher the hereditary risk of cancer development, the easier it is for any environmental factor of even the slightest risk to trigger tumor formation.

**Table 1:** Studied genes and polymorphisms

Gene	Latin name	Polymorphism	dbSNP assigned reference SNP ID	Locus	Gene functions
CytochromeP450 1А1	*CYP1A1*	T606G	rs2606345	15q24.1	The 1-st phase of detoxification - metabolic activation of the aromatic hydrocarbons
		A4889G Ile462Val	rs1048943		
Glutathione S-transferase mu 1	*GSTM1*	Insertion-deletion	-	1p13.3	The 2-nd phase of detoxification – detoxification proper by conjugation of reduced glutathione to a wide number of exogenous and endogenous hydrophobic electrophiles
Glutathione S-transferase theta 1	*GSTT1*	Insertion-deletion	-	22q11.2	
Glutathione S-transferase mu 3 (brain)	*GSTM3*	G670A V224I	rs7483	1p13.3	
Glutathione S-transferase pi 1	*GSTP1*	A313G Ile105Val	rs1695	11q13	
X-ray repair, complementing defective, in chinese hamster, 1	*XRCC1*	C589T Arg194Trp	rs1799782	19q13.2	Base excision repair
Excision-repair, complementing defective, in chinese hamster, 2	* ERCC2 (XPD) *	A2251C Lys751Gln	rs13181	19q13.3	Nucleotide excision repair
		G862A Asp312Asn	rs1799793		
8-oxoguanine-DNA-glycosylase	*OGG1*	C977G Ser326Cys	rs1052133	3p26.2	Base excision repair - removal 8-oxodeoxyguanosine
Nitric oxide synthase, neuronal	*nNOS (NOS1)*	C276T	rs2682826	12q24.2	NO production in neuronal tissues
5,10-methylenetetrahydrofolate reductase	*MTHFR*	C677T Ala222Val	rs1801133	1p36.3	Conversion of 5,10-methylene­tetra­hydrofolate to 5-methyl­tetrahydrofolate, a cosubstrate for homocysteine remethylation to methionine

Despite the fact that 20% of all the solid tumors in children are brain tumors, there have only been several associative studies of brain tumors on children from various ethnic populations. In a cohort of 73 children in Thailand with various types of CNS tumors it was demonstrated an increased number of homozygous carriers of the minor variant of the *MTHFR * gene (polymorphism A1298C), which is involved in folate metabolism [20]. A study in the United States analyzed the distribution of xenobiotic detoxification gene alleles of *GSTM1 * (insertion/deletion) *, GSTT1 * (insertion/deletion), and  *GSTP1 * (Ala114Val) genes among 173 child patients and registered the association of a functional allele of *GSTM1 * and a rare genotype of *GSTP1 * (Val114/Val114) with pediatric astrocytoma [21]. The same researchers showed that a combined cohort of adults (92) and children (43) with brain tumors displayed a distribution of Arg72Pro genotype frequencies for the *P53* gene that was considerably different from the control group. It has also been reported that highly malignant astrocytoma patient cohorts exhibit an increased number of heterozygous individuals for this *P53* gene polymorphism [22].

Interaction of the environment and the genotype in relation to brain tumor incidence in childhood has been analyzed in two studies [23, 24]. In the case of exposure to phosphoorganic insecticides *in utero* or after birth, the increased risk of developing brain tumors is significantly associated with a polymorphism of the *PON1 * (C108T) detoxification gene, for which the above-said compounds are a substrate [23]. This study moved on to confirm the effects of *PON1 * on a larger cohort (201 people) and also showed associationswith the risk of brain tumor developmentfor two other detoxification genes involved in insecticide metabolism, *FMO1 * (C9536A) and  *BCHE * (A539T) [24].

**Table 2:** Age-specific mortality in Russia in the 0-24 *-* year agerange

Age, years	Deaths per 1000 population		
	2006	2007	2008
0	10.2	9.4	8.5
1-4	0.7	0.6	0.6
5-9	0.4	0.3	0.3
10-14	0.4	0.4	0.3
15-19	1.1	1.1	1.1
20-24	2.2	2.1	1.9

This study presents the results of an associative study of genetic risk factors related to the formation of brain tumors in children. The choice of genotyping loci was based on literary data and on personal results obtained in a study of susceptibility genes that increase somatic mutability [25]. This study also includes genes which are primarily expressed in the brain ( *GSTM3* aka brain * GSTM* ) and in neural tissues ( *NOS1,* or  *nNOS* – neuronal) and which exhibit association with some oncological diseases [26, 27]. The involved loci are described in Table 1.

##  Experimental procedures 

A cohort of 172 children with malignant CNS tumors (92 boys and 80 girls) aged 2-16 were included in this study. These children were under treatment in the laboratory of the Children’s X-ray Radiology of the Russian Scientific Center ofRoentgenoradiology from 2007 to 2010. The average age of the child patients was 8.96±0.38. The most common tumors in the studied cohort were medulloblastomas ( *N* = 58) and brain stem tumors ( *N* = 26). Apart from these, there were also cases of apoplastic ependymoma ( *N* = 19), glioblastoma ( *N* = 10), germinogenic tumors ( *N* = 6), low malignancy astrocytoma ( *N* = 5), high malignancy astrocytoma ( *N* = 5), primitive neuroectodermal tumors ( *N* = 5), and others ( *N* = 38). The control group consisted of 183 people (102 males and 81 females) aged 17 to 21, an average age of 19.90 ± 0.08 years. All the sick children and youths from the control group were of Caucasian race. The patient database contains information on their places of birth and residence. The children’s parents gave informed consent for the genotyping procedure. The ten-year difference in the average age of the patient and control groups could not have any significant effect on the allelic variant frequencies in the groups, since mortality in this age group does not exceed 0.1% (Table 2) [28]. Moreover, the first four main causes of death in the 15–24-age group are violence-related: unintentional bodily harm, suicide, undefined bodily harm and murder [29]. The criteria for involvement into the control group were age, nationality, birthplace inside the central regions of the European territory of the Russian Federation, and informed consent to the procedures.

DNA was extracted from peripheral blood lymphocytes using a Diatom DNA Prep 200 kit, which uses guanidine isocyantate and Nucleus–sorbent (Isogen Laboratory, Russia). Genotyping was performed using allele-specific tetraprimer PCR [30]. This method allows the amplification of DNA fragments of alternative alleles in a single test tube. The amplification products were separated using agarose gel electrophoresis and then stained with ethidium bromide.

 The statistical analysis was performed using standard methods available in the WinSTAT 2003.1 software integrated into Microsoft Excel. 

 Estimation of the odds ratios (OR) and the significance of the odds ratio according to the precise Fischer test was accomplished using the free-use software WinPepi: http://www.brixtonhealth.com/pepi4windows.html. 

##  Results 

 We identified the genotypes of the studied individuals at 12 polymorphic sites of 10 genes. The genotype frequencies in the control group and the patient group were in accordance with the Hardy-Weinberg distribution. 

Table 3 compares the frequencies of allele and genotype occurrence for 12 polymorphic sites in children with various tumors of the CNS, as well as youths in the control group. We also distinguished two major groups in the child patients cohort - a group with medulloblastoma and a group with brain stem tumors.

**Table 3:** Genotypes frequencies among the brain tumors patients and in the control group

Loci, alleles, genotypes		Frequencies (%)			
		All brain tumors (N*=172)	Medulloblastoma (N*=63)	Brainstem tumor (N*=26)	Healthy (N*=183)
*CYP1A1 *T606G rs2606345	T	187 (54.68)	67 (53.18)	30 (57.69)	236 (64.48)
	G	155 (45.32)	59 (46.83)	22 (42.31)	130 (35.52)
	T/T	57 (33.33)	22 (34.92)	10 (38.46)	78 (42.62)
	T/G	73 (42.69)	23 (36.51)	10 (38.46)	80 (43.72)
	G/G	41 (23.98)	18 (28.57)	6 (23.08)	25 (13.66)
*CYP1A1 *A4889G rs1048943	A	329 (95.64)	120 (95.24)	49 (94.23)	352 (96.18)
	G	15 (4.36)	6 (4.76)	3 (5.77)	14 (3.83)
	A/A	157 (91.28)	57 (90.48)	23 (88.46)	169 (92.35)
	A/G	15 (8.72)	6 (9.52)	3 (11.54)	14 (7.65)
	G/G	0 (0.00)	0 (0.00)	0 (0.00)	0 (0.00)
*GSTM1*	D/D	93 (54.07)	35 (55.56)	16 (61.54)	95 (51.91)
	I/*	79 (45.93)	28 (44.44)	10 (38.46)	88 (48.09)
*GSTT1*	D/D	45 (26.16)	20 (31.75)	9 (34.62)	28 (15.30)
	I/I*	127 (73.84)	43 (68.25)	17 (65.38)	155 (84.70)
* GSTP1 *A313G rs1695	A	242 (70.35)	87 (69.05)	31 (62.00)	247 (67.49)
	G	102 (29.65)	39 (30.95)	19 (38.00)	119 (32.51)
	A/A	80 (46.51)	29 (46.03)	8 (32.00)	79 (43.17)
	A/G	82 (47.67)	29 (46.03)	15 (60.00)	89 (48.63)
	G/G	10 (5.81)	5 (7.94)	2 (8.00)	15 (8.20)
* GSTM3 *G670A rs 7483	G	203 (59.01)	80 (63.49)	28 (53.85)	222 (60.66)
	A	141 (40.99)	46 (36.51)	24 (46.15)	144 (39.34)
	G/G	63 (36.63)	26 (41.27)	8 (30.77)	73 (39.89)
	G/A	77 (44.77)	28 (44.44)	12 (46.15)	76 (41.53)
	A/A	32 (18.60)	9 (14.29)	6 (23.08)	34 (18.58)
* NOS1 *C276T rs 2682826	C	243 (70.64)	90 (71.43)	33 (63.46)	271 (75.70)
	T	101 (29.36)	36 (28.57)	19 (36.54)	87 (24.30)
	C/C	84 (48.84)	33 (52.38)	9 (34.62)	103 (57.54)
	C/T	75 (43.60)	24 (38.10)	15 (57.69)	65 (36.31)
	T/T	13 (7.56)	6 (9.52)	2 (7.69)	11 (6.15)
* MTHFR *C677T rs1801133	C	228 (70.81)	76 (66.67)	36 (72.00)	221 (67.79)
	T	94 (29.19)	38 (33.33)	14 (28.00)	105 (32.21)
	C/C	80 (49.69)	25 (43.86)	13 (52.00)	70 (42.94)
	C/T	68 (42.24)	26 (45.61)	10 (40.00)	81 (49.69)
	T/T	13 (8.07)	6 (10.53)	2 (8.00)	12 (7.36)
* XRCC1 *C589T rs 1799782	C	322 (93.61)	119 (94.44)	46 (88.46)	337 (94.13)
	T	22 (6.40)	7 (5.56)	6 (11.54)	21 (5.87)
	C/C	150 (87.21)	56 (88.89)	20 (76.92)	160 (89.39)
	C/T	22 (12.79)	7 (11.11)	6 (23.08)	17 (9.50)
	T/T	0 (0.00)	0 (0.00)	0 (0.00)	2 (1.12)
* XPD *T2251G rs 13181	T	212 (61.63)	82 (65.08)	32 (61.54)	248 (68.13)
	G	132 (38.37)	44 (34.92)	20 (38.46)	116 (31.87)
	T/T	63 (36.63)	25 (39.68)	9 (34.62)	84 (46.15)
	T/G	86 (50.00)	32 (50.79)	14 (53.85)	80 (43.96)
	G/G	23 (13.37)	6 (9.52)	3 (11.54)	18 (9.89)
* XPD *G862A rs1799793	G	211 (61.70)	82 (66.13)	32 (61.54)	242 (66.48)
	A	131 (38.30)	42 (33.87)	20 (38.46)	122 (33.52)
	G/G	64 (37.43)	26 (41.94)	9 (34.62)	80 (43.96)
	G/A	83 (48.54)	30 (48.39)	13 (50.00)	82 (45.05)
	A/A	24 (14.04)	6 (9.68)	4 (15.38)	20 (10.99)
* OGG1 *C977G rs1052133	C	274 (80.59)	92 (77.97)	40 (76.92)	270 (78.04)
	G	66 (19.41)	26 (22.03)	12 (23.08)	76 (21.97)
	C/C	116 (68.24)	36 (61.02)	18 (69.23)	105 (60.69)
	C/G	42 (24.71)	20 (33.90)	4 (15.38)	60 (34.68)
	G/G	12 (7.06)	3 (5.08)	4 (15.38)	8 (4.62)

*The number of individuals genotyped at certain loci may differ.

Note: Genotypes associated with diseases are highlighted in grey.

In cases where the polymorphism was of an insertion/deletion nature (genes *GSTM1, GSTT1* ), we compared two genotypes: “zero” – homozygous deletion (D/D) and “functional” – a genotype with a functional allele in either homo- or heterozygous form (I/∗). Hence and further ∗ depicts an unspecified allele.

Increased susceptibility to brain tumor development was observed for carriers of the D/D genotype of the *GSTT1 * gene. A two-side Fischer test for all the CNS tumor types yields *p* = 0.013, OR = 1.96, 95% confidence interval 1.16–3.32; for medulloblastoma patients - *p* = 0.009, OR = 2.57, 95% confidence interval 1.33–4.99; for children with brain stem tumors - *p* = 0.026, OR = 2.93, 95% confidence interval 1.21–7.12. Of all the analyzed two-loci combinations, the one associated with the highest risk of malignant brain tumors turned out to be a double deletion of *GSTM1-GSTT1* (27 people – 15.7% patients; *p* = 0.017, OR = 2.42, 95% confidence interval 1.18–4.95) (Figure).

**Fig. 1:**
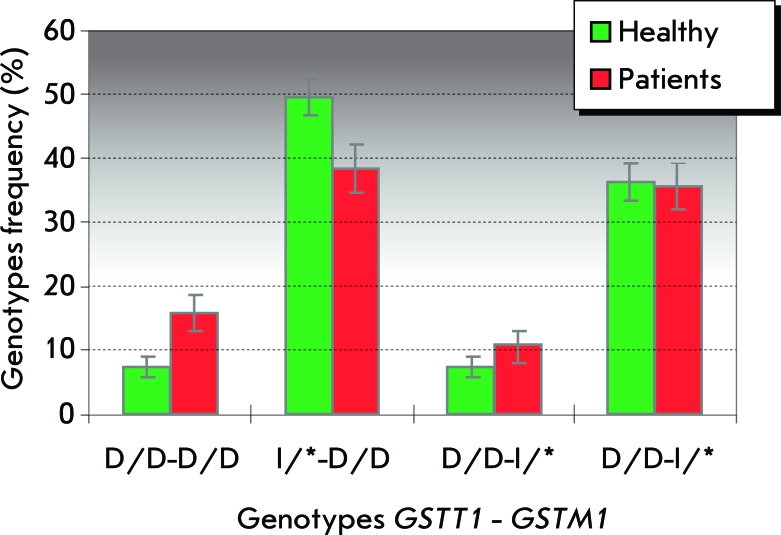
Frequencies of *GSTT1-GSTM1* allele combinations among children with malignant brain tumors and in the healthy group.

The risk of developing any type of brain tumor, and specifically a medulloblastoma, turned out to be up in carriers of the minor 606G allele of the *CYP1A1* gene (for all types of tumor - *p* = 0.009, according to the two-sided Fischer test, OR= 1.50, 95% confidence interval 1.11–2.03, for medulloblastoma - *p* = 0.026, OR = 1.60, 95% confidence interval 1.06–2.41).

Among the brain stem tumor patients there was an elevated number of *NOS1* *276T minor allele carriers in both homo- and heterozygous forms, where *p* = 0.035, OR = 2.56, 95% confidence interval 1.10–5.96. The whole patient group also exhibited an increased occurrence of the minor allele; however, these data were statistically insignificant - *p* = 0.11, OR = 1.42, 95% confidence interval 0.93–2.16.

There was also a tendency for association between the 2251G minor allele of the nucleotide excision DNA repair gene *XPD* in both homo- and heterozygous forms and an increased chance of developing a brain tumor ( *p* = 0.084, OR = 1.48, 95% confidence interval 0.97–2.27).

##  Discussion 

The detoxification of xenobiotics consists of two main stages of detoxification and a third stage - secretion of the detoxified products out of the cell. The first stage involves activation of the xenobiotic compounds by P-450 cytochromes and a number of other enzymes. The second stage is the detoxification, *per se,* and it involves glutathione-S-transferases and other proteins. The intermediary electrophilic metabolites that were formed in the first stage are toxic, and effective detoxification requires a fine balance between the activity of the first- and second-stage enzymes. This balance is deregulated both by insufficient activity of the polymorphic variants of the second-stage enzymes and by the increased activity of the first-stage enzymes [31]. Increased activity of the first-stage detoxification enzymes and insufficient activity of the second-stage enzymes (GST) cause an increase in the level of activated electrophilic metabolites, thus increasing the deleterious effects of the xenobiotic compounds.

This study demonstrates that there is an association between certain xenobiotic detoxification gene alleles and the development of brain tumors in children. The risk of developing malignant tumors in the brain during childhood is increased in carriers of a minor variant of *CYP1A1* (606G).

The role of *CYP1A1* polymorphism has not been studied in relation to child neurooncology. Associative studies on adults have shown no association between *CYP1A1* A4889G (Ile462Val) polymorphism and the risk of developing glioma or several other types of malignant brain tumors [32–34]. The *CYP1A1* gene is located in the 15q22-24 region, and people with a hereditary predisposition towards glioma have exhibited associations between the disease and low-penetrance markers in the 15q23-q26.3 region which overlaps this locus [35].

Data on the role of *CYP1A1* gene polymorphism in carcinogenesis are contradictory, and it seems that their role considerably depends on the interaction between the genotype and the environment [36]. The T606G site is located in the first intron of the *CYP1A1 * locus. The single nucleotide substitutions (SNP) located in the intron regions do not usually influence gene activity. However, the T606G polymorphism has been associated with lung cancer [37], hormone-dependent tumors [38], and with the level of sex hormones, which are substrates of CYP1A1 [39]. There are data on the T606G site which indicate that in the absence of specific substrates, the allelic 606T (SNP T606G) variant of the *CYP1A1 * gene is expressed more actively, whereas the 606G variant is induced in the presence of specific substrates (polycyclic aromatic hydrocarbons of exogenous origins, such as foods, industrial waste, tobacco smoke, as well as endogenous compounds, such as estrogens). The differential effect of the allelic variants 606G and 606T on the observed effects under ecologically unfavorable conditions (industrial pollution of air, smoking), as well as in their absence, has been demonstrated in two independent studies [40, 41]. The two studied sites in the *CYP1A1 * locus which were studied in this work arein strong linkage disequilibrium - D’=0.913, r=0.229, p=0, and the minor alleles (4889G, 606G) belong to a single linkage group. Linkage disequilibrium data in the patient and control groups were identical. In this work, we have confirmed our previous data obtained on different cohorts and indicating that the polymorphic sites A4889G and T606G are linked [25, 42]. According to data from HapMap, the frequency of the 606G allele in Caucasians is 0.36–0.45, while the frequency of 4889G is 0.04–0.07. Until recently, researchers had studied three major polymorphic sites in the *CYP1A1 * gene in European populations: T3801C, A4889G, and C4887A [43]. Besides, polymorphism T606G has a functional character, the frequency of the 606G allele is higher than the frequency of the minor alleles at other polymorphic sites. Thus, this allele seems to be a new promising marker for associative studies of multifactor diseases.

Our study also shows associations between the formation of malignant brain tumors and the possession of deletion variants of *GSTT1* (D/D) (OR=1.96, *p* = 0.013). Association between polymorphism of glutathione-S-transferase genes, which encode enzymes for the second phase of xenobiotic detoxification, and the development of brain tumors in children, was analyzed in study [21]. Statistically significant results were obtained for the functional allele of *GSTM1* and a minor allele of *GSTP1 * 313G *. * Association between the development of malignant brain tumors in adults and polymorphisms of glutathione-S-transferase-encoding genes was analyzed much more thoroughly; for instance, 10 studies were conducted in Europe [32–34, 44–50]. The results of seven of these studies and the results of the aforementioned work on a cohort of sick children [21] were combined in a meta-analysis [51], which was performed for two of the most common nosological forms: gliomas and meningiomas. According to this meta-analysis, in Caucasians the deletion variant of *GSTT1* occurred significantly more often in meningioma patients (OR = 1.95). No differences in the frequencies of the *GSTM1 * (Ins/Del)and  *GSTP1* A313G (Ile105Val) and the adjacent C341T (Ala114Val) allele were observed between the patient and control groups. Another large-scale study obtained data indicating the absence of an association between polymorphisms of *GSTM1 * (Ins/Del), * GSTM3 * (A63С), * GSTT1* (Ins/Del) and the development of gliomas, glioblastomas, and meningiomas. It was demonstrated that the 105G-114C (Val-Ala) haplotype of *GSTP1* has a weak protective effect on the chance of developing glioma [32].

No significant differences in DNA repair gene allele frequencies were found (Table 3); however, there was a tendency level association of the minor 2251G allele in the *XPD* locus with an elevated chance of developing the disease.

Associative studies of malignant tumors of any localization concerning DNA repair gene polymorphisms most often involve *XPD* nucleotide excision repair genes and *XRCC1 * base excision repair genes[52], which are located on the same region of the chromosome (19q13.2–3). Most of the results of associative studies of brain tumors are summarized in review [53]. It was shown that in adults, the most common malignant tumors of neuroepithelial tissues are associated with the nucleotide excision repair genes *XPD, ERCC1* and a gene located in the same (19q13.2-3) region of the chromosome - *GLTSCR1* (glioma tumor suppressor candidate of an unknown function) [54]. Caggana *et al* . [55] showed that of 7 polymorphic sites in the *XPD * gene, maximum association with an increased risk of glioma was observed for the least studied synonymous Arg156Arg polymorphism, which may be a marker of another unknown gene that predisposes potential patients to this disease. Sites T2251G (Lys751Gln) and G862A (Asp312Asn) of the *XPD* gene are located 12340 b.p. apart and are linked. This work has obtained the following linkage disequilibrium data: D’=0.674, r=0.662, p=0 (no difference between the patient and control groups), which is in agreement with the published data on Caucasians [56]. Despite the absence of significant results concerning DNA repair genes in this work, studying polymorphic loci in the 19q13.2-3 chromosome region seems a promising line of research that could lead to the discovery of risk markers for malignant brain tumors in children.

This study also shows that a minor allele of the neuronal nitric oxide synthase occurs significantly more often in patients with brain stem tumors (Table 3); differences for the whole patient group are statistically insignificant.

Genes from the nitric oxide synthase family, which includes the neuronal nitric oxide synthase gene, are usually studied in connection with inflammatory processes. However, *nNOS * polymorphism is associated with melanoma predisposition [27]. Melanoma is included into the nerve-ending tumor group. Families that have a hereditary predisposition towards brain tumors are often predisposed towards developing melanomas as well [5]. Taking into account the elevated expression of *nNOS* in nervous tissue, as well as the putative cross-sensitivity to melanoma and glioma, we resolved to analyze the C276T site of the *nNOS * gene. This polymorphism is considered to be functional, since the single nucleotide substitution in the untranslated region results in elevated mRNA expression of the minor variant [57]. Significant results on the association of the minor allele with increased risk of developing brain tumors were only observed for the small group of patients with brain stem tumors and will of course require further study.

Our previous associative studies of xenobiotic detoxification genes have shown that women with reproductive system diseases (mainly myomas and chronic cystic mastitis) carrying the 606G, 4889G alleles of the *CYP1A1* gene have an increased frequency of somatic mutations at the T-cell receptor (TCR) locus in peripheral blood lymphocytes (phentoype CD ^3-^ CD ^4+^ ). It is known that the number of such TCR-mutant lymphocytes is elevated in cancer patients (cancer of the larynx and hypopharynx, thyroid gland tumor, cervical cancer and Hodgkin’s lymphoma) and in people with hereditary predispositions towards oncological diseases (ataxia-teleangiectosia) [58, 59]. The single direction of the effects in two separate studies may indicate the pleiotropic effect of detoxification genes, which leads to insufficient resistance of the organism in the genotype-environment interaction process. Besides the possible increased risk of disease due to altered detoxification enzyme activity, allelic variants associated with somatic mutability and with predisposition to the formation of malignant tumors in childhood may act as markers of a linked group of unknown genes that can be responsible for some of the observed effects. The obtained results, if confirmed by independent studies, can be useful for identifying the genetic risk factors involved in the formation of malignant tumors in children.
